# Ranolazine Toxicity Secondary to Paxlovid

**DOI:** 10.7759/cureus.37153

**Published:** 2023-04-05

**Authors:** Bradley Casey, Robert C Vernick, Amol Bahekar, Divyang Patel, Inmaculada Ncogo Alene

**Affiliations:** 1 Internal Medicine, Cape Fear Valley Medical Center, Fayetteville, USA; 2 Cardiology, Cape Fear Valley Medical Center, Fayetteville, USA

**Keywords:** cad: coronary artery disease, altered mental status evaluation, ranolazine, covid 19, paxlovid

## Abstract

An Emergency Use Authorization (EUA) was issued by the FDA on December 22, 2021 for the investigational antiviral drug nirmatrelvir copackaged with the HIV-1 protease inhibitor ritonavir (Paxlovid - Pfizer) for outpatient treatment of mild to moderate COVID-19 in children 12 years and old that are high risk of severe disease. Due to the effects, Paxlovid has on liver metabolism it has a copious amount of drug-to-drug interactions. Here we present a rare case of a patient that was given Paxlovid and continued to take her Ranolazine at home. She presented to the emergency department obtunded and after an initial workup, it was determined to be secondary to ranolazine toxicity. She eventually recovered over 54 hours and returned to her baseline.

## Introduction

Coronavirus is classified as a single positive-stranded, enveloped RNA virus, and severe acute respiratory syndrome coronavirus 2 (SARS-CoV-2) is a type with high pathogenicity [1]. SARS-CoV-2 has created one of the worst pandemics of the 21st century in the form of coronavirus disease 2019 (COVID-19) [1]. In the wake of the COVID-19 pandemic, the prevention of severe disease remains a top priority for public health [2]. The Food and Drug Administration (FDA) approved Paxlovid (ritonavir-boosted nirmatrelvir) for its efficacy and approval to prevent hospitalization and mortality in adults with mild-to-moderate COVID-19. [2]. As part of Paxlovid, nirmatrelvir, a new protease inhibitor that targets the 3CLpro of SARS-CoV-2, is combined with ritonavir, which inhibits cytochrome P450 3A4 and increases nirmatrelvir's serum levels [3]. Paxlovid should be used with caution with any patient with coronary artery disease (CAD) receiving cardiovascular medications due to the risk of adverse medication reactions [2]. We are presenting a rare case where a patient with CAD on Ranolazine (Ranexa) was prescribed Paxlovid for her mild COVID-19 symptoms and developed Ranexa toxicity.

## Case presentation

**Figure 1:**
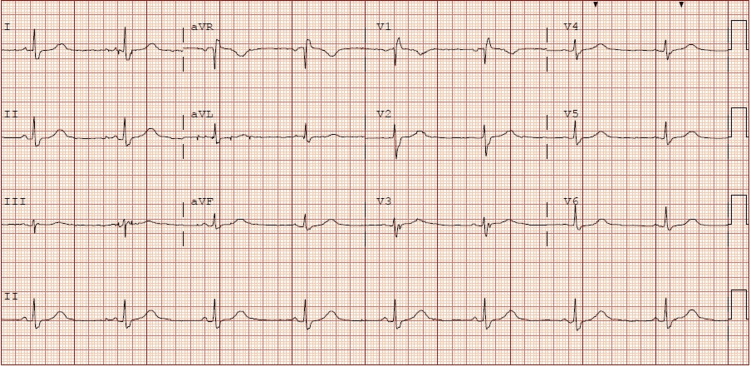
Sinus rhythm 48 beats per minute with a right bundle branch block.

A 70-year-old female patient with a past medical history of type 1 diabetes, hypertension, coronary artery disease (coronary artery bypass grafting seven years ago), and chronic kidney disease stage II presented from home by emergency medical services (EMS) to the emergency department with altered mental status. She initially went to her primary care provider (PCP) six days ago with complaints of fatigue, generalized weakness, non-productive cough, and muscle aches. The patient was diagnosed with COVID-19 in the office and her PCP prescribed Paxlovid. The husband reported that she was on day 3 of Paxlovid when she started to become slightly confused and not acting herself, but the family thought it was all due to her COVID-19 infection. She completed all five days of Paxlovid, and on the evening of the fifth day, she was completely altered and non-vocal. At this time husband confirmed she had taken Ranexa and atorvastatin with Paxlovid all five days. The husband reported that all he could do was to get her to open her eyes. At this time, the husband called EMS, and when they arrived the patient oxygen saturation was 80% on room air and she was placed on a 2-liter nasal cannula. Once the patient arrived at the Emergency department her vitals were 97.9 degrees Fahrenheit, heart rate was sinus bradycardia 52 beats per minute, her respiratory rate 15, her blood pressure 166/66, and her oxygen saturation 95% on 2 liters oxygen. Initial blood work can be seen in Table 1 and home medications in Table 2. Electrocardiogram (EKG) in Figure 1 shows sinus rhythm at 48 beats per minute with a right bundle branch block.

**Table 1:** Initial blood work when patient arrived to the emergency department.

Laboratory Test	Reference Range	Patient's Lab
Complete Blood Count		
White Blood Cell Count	4.5 - 12.5 x10*3/µL	10.4x10*3/µL
Hemoglobin	12.0 - 16.0 g/dL	10.7 g/dL
Mean Corpuscular Volume	81.0 - 99.0 fL	90.9 fL
Platelets	150 - 450 x10*3/µL	214x10*3/µL
Comprehensive Metabolic Panel		
Sodium	136 - 145 mmol/L	132 mmol/L
Potassium	3.5 - 5.1 mmol/L	4.7 mmol/L
Bicarbonate	21 -32 mmol/L	25 mmol/L
Chloride	98 - 107 mmol/L	96 mmol/L
Blood Urea Nitrogen	7 - 25mg/dL	18 mg/dL
Creatinine	0.60 mg/dL	1.06 mg/dL
Glucose	74 - 106 mg/dL	139 mg/dL
aspartate aminotransferase	15 - 37 U/L	21 U/L
alanine transaminase	12 - 78 U/L	14 U/L
glomerular filtration rate	>60.0 mL/min/1.73m*2	56.6 mL/min/1.73m*2
Arterial Blood Gas		
Arterial pH	7.35 - 7.45 pH	7.41 pH
Carbon Dioxide	35.0 - 45.0 mm Hg	39 mm Hg
Bicarbonate on Arterial Blood Gas	22.0 - 26.0 mEq/L	24 mEq/L
Urinalysis		
Urine Leukocytes	Negative	Negative
Urine Nitrites	Negative	Negative
Urine Bacteria	None Seen	None Seen
Urine White Blood Cell Counts	0-10/high powered field	6 high powered field
Other Blood Tests		
Ammonia	11 - 32 µmol/L	27 µmol/L
Thyroid Stimulating Hormone	0.358 - 3.740 µIU/mL	3.874 µIU/mL
Free T4	0.76 - 1.46 ng/dL	1.47 ng/dL
Ethanol Level	<3 mg/dL	<3 mg/dL
High Sensitivity Troponin	2 - 15 pg/mL	14 pg/mL
3 hour Repeat High Sensitivty Troponin	2 - 15 pg/mL	12 pg/mL
Creatinine Phosphokinase	26 - 192 U/L	117 U/L
Sedimentation Rate	0 -30 mm/hr	22mm/hr
C-reactive Protein	< 5 mg/L	52 mg/L

Due to her altered mental status CT scan of the head without contrast was performed and showed no acute intracranial abnormality. CT scan of the chest with and without contrast was done to rule out pulmonary embolism due to her recent history of COVID-19 and hypoxia, and results showed no evidence of central pulmonary embolism. At this time, poison control was called with concern of Ranexa toxicity due to consumption of Ranexa and Paxlovid simultaneously. Poison control recommended supportive therapy because Ranexa is too highly protein bound to be dialyzed off. The patient was admitted to the floor for monitoring. The patient remained hemodynamically stable throughout hospitalization and was eventually transitioned to room air. After approximately 54 hours she returned to her baseline. The patient was then discharged to subacute nursing facility to complete physical therapy.

**Table 2:** List of the patient's home medications.

Home Medication	Dose	Frequency
Amlodipine	10mg	Daily
Aspirin	81mg	Daily
Atorvastatin	80mg	Nightly
Clopidogrel Bisulfate	75mg	Daily
Dapsone	100mg	Daily
Ferrous Sulfate	325mg	Daily
Fexofenadine	180mg	Daily
Folic acid	1mg	Daily
Furosemide	20mg	Twice Daily
Gabapentin	300mg	Three times daily
Isosorbide Mononitrate	120mg	Daily
Insulin Glargine	35 units	Nightly
Losartan	50mg	Daily
Ranolazine	1000mg	Twice Daily

## Discussion

As a treatment for chronic stable angina, ranolazine was approved by the FDA in 2006 [4]. In the combination assessment of ranolazine in stable angina trials (CARISA), ranolazine was shown to reduce angina attacks on a weekly basis [5]. As a therapeutic agent, it inhibits late-phase sodium channels in ischemic cardiac myocytes, which reduces intracellular sodium concentration and, therefore, intracellular calcium influx through Na-Ca channels [4]. Ranolazine helps with angina due to when oxygen consumption is reduced, and intracellular calcium is reduced [4]. CYP3A4 and CYP2D6 enzymes are primarily responsible for metabolizing ranolazine in the liver [4]. The metabolism of ranolazine in this case is important due to the fact that Paxlovid inhibits cytochrome P450 3A4 [3].

The most common side effects associated with ranolazine use are headaches (5.5%), dizziness (1% to 6%), constipation (5%), and nausea (≤4%; dose-related) [6]. When ranolazine is taken in high doses, it can cause nausea, vomiting, dizziness, tremors, dysphagia, hallucinations, unsteady gait, and nausea [4]. Ranolazine is also known to cause a dose-dependent increase in the QT interval. [6]. Very few case reports have been published in regard to Ranolazine overdose. In Ranolazine the four most prevalent metabolites in plasma have elimination half-lives of six to 22 hours [7]. Just as poison control informed us, an overdose of ranolazine is not effectively cleared by hemodialysis because it is 62% bound to plasma proteins [4].

Nirmatrelvir/ritonavir received an Emergency Use Authorization (EUA) from the FDA on December 22, 2021 for treating COVID-19 [8]. When symptoms begin, patients should start taking Nirmatrelvir 300 mg twice daily for five days, accompanied by ritonavir 100 mg twice daily [8]. Currently the FDA has Ranolazine listed under contraindicated when taking with Paxlovid due to serious and life-threatening reactions [8]. When she initially arrived to the hospital her bradycardia that was seen on the EKG could have been from the Paxlovid or Ranexa because they both have been known to cause bradycardia [4,8}. Currently, supportive care is the only treatment option for patients that present with the concern for Ranolazine toxicity [2,4]. Patients should be monitored with electrocardiogram and telemetry due to Ranolazines ability to prolong QT interval [4,5]. The half-life as reported could be anywhere from 6 to 22 hours, but our patient did not return to her baseline until 54 hours [7].

## Conclusions

Here we present an interesting case of a patient that took Paxlovid with Ranolazine which resulted in Ranolazine toxicity. With the patient's initial presentation, innumerable differentials could be crossing a physician's mind. It is important to always go over the patient's home medications and any new additional medications. It is also important to warn patients about potential drug side effects and interactions when prescribing new medications. The FDA currently has a prescriber sheet to review before prescribing this medication to patients to avoid any unnecessary cross-drug reactions. Our patient and family initially thought that her confusion was part of the viral symptoms that come with COVID-19 and never thought about potential drug interactions.
